# Analysis of Spanish Parents’ Knowledge about ASD and Their Attitudes towards Inclusive Education

**DOI:** 10.3390/ejihpe12070063

**Published:** 2022-07-21

**Authors:** Irene Gómez-Marí, Raúl Tárraga-Mínguez, Gemma Pastor-Cerezuela

**Affiliations:** 1 Department of Education and School Management, Faculty of Teacher Training, University of Valencia, 46022 Valencia, Spain; raul.tarraga@uv.es; 2 Department of Basic Psychology, Faculty of Psychology and Speech Therapy, University of Valencia, 46010 Valencia, Spain; gemma.pastor@uv.es

**Keywords:** Asperger’s, attitudes, autism, family, inclusion, knowledge

## Abstract

To make possible the inclusion of children with autism spectrum disorder (ASD) in mainstream settings, parental knowledge and attitudes towards the disorder play a key role between the home and the school setting. However, prior literature has not carried out an in-depth analysis of parents’ knowledge about ASD and their attitudes toward the inclusion of children with this diagnosis. This study examined the parental attitudes towards inclusion and knowledge about ASD. Participants were parents of children with ASD (*n* = 75), parents of children without ASD whose children had prior or current contact with peers with ASD (*n* = 44), and parents of children with no previous interactions with a peer with ASD (*n* = 51). The Attitudes of Regular Educators Towards Inclusion for Students with Autism Survey and the Autism Knowledge Questionnaire were filled out. Nonparametric statistical tests were used. Results showed that parents of children with ASD have better knowledge about this disorder and hold more favorable attitudes towards the inclusion of children with ASD than the other parents. These findings suggest that the benefits of inclusive schooling are limited to the school setting and do not appear to affect families of children without ASD.

## 1. Introduction

### 1.1. Autism Spectrum Disorder in Mainstream Schools

Autism spectrum disorder (ASD) is a neurodevelopmental condition that affects social interactions and communication skills. Children with ASD show repetitive behavior patterns and focused interests. The way people with ASD process and perceive the world around them is also affected [1].

From 2000 to the present, the prevalence of ASD has increased notably, and it is estimated to be around 1/100 [2]. In Spain, recent educational reforms have proposed inclusive schooling models. Thus, it is increasingly common for students with ASD to attend school in mainstream settings.

Although ASD rates are quite high, and many people probably have a connection to ASD, either personally, in educational environments, or even in the workplace [3], stigma can still occur [4,5,6]. In educational settings, children with challenging behaviors, as in ASD, are less likely to be accepted by their typically developing peers [5,7,8].

The stigmatization is subordinated to the core ASD characteristics [9]. People with ASD can have maladaptive behavior, even though they show a normal physical appearance [10]. In addition, some studies [11,12] found that stigma towards families of children with ASD was higher than stigma toward families of children with intellectual or physical disabilities.

Thus, it is likely that every child with a disability enrolled in mainstream schools frequently faces a lot of barriers. One of the most difficult barriers is a stigmatized social image of disability, prejudices, and negative attitudes. Stigma in the educational environment can be caused by teachers, parents, and/or peers, and it depends on cultural factors [9]. The inclusive education model thus involves the entire educational community: not only teachers or peers, but also families with or without children with disabilities [13].

Children’s conceptions about other people, including individuals with disabilities, are probably influenced by the significant adults (parents, caregivers, teachers) in their lives [14]. In daily interactions, adults display their own values, transferring knowledge and attitudes to their children and influencing their children’s conceptions [15,16,17,18,19]. Their support is considered essential in facilitating inclusive educational practices [20,21,22]. 

### 1.2. Parental Attitudes and Knowledge about ASD

Attitudes are described as beliefs, feelings, and behavioral tendencies toward socially significant issues or situations [23,24], such as including students with ASD in mainstream schools. Knowledge about ASD refers to general information such as the core symptoms of ASD, the possibilities for treatment, and the etiology of this disorder.

Parents are usually children’s first caregivers and a key link between the home and school settings [25]. Because children learn from their significant adults, it would be useful to assess parents’ attitudes and knowledge about the inclusion of children with ASD, not only in families of children with ASD, but also in families of children without ASD.

Parents’ attitudes towards inclusion appear to be varied, complex, and multidimensional, and they may be affected by a wide range of factors [26,27,28]. 

On the one hand, some parents of children with high support needs do not favor inclusion and prefer that their children attend special education schools [16]. These parents are concerned about the suitability of mainstream classrooms for their children: they are worried that their children could be mistreated, harmed, or ridiculed. They also contend that regular teachers have inadequate training and attitudes and lack the necessary support and resources [29,30,31]. Moreover, having a child with ASD is related to high levels of stress [6,32] and stigma [9]. Therefore, families of children with ASD have concerns about the effective inclusion of their children.

On the other hand, some families show positive attitudes towards inclusion as a way to improve the behavioral, social, and language skills of their children with ASD [29,33,34,35]. Some previous research involving parents of children with disabilities also confirmed that inclusion can benefit the development of children with any kind of diagnosis [8,33,34].

However, information about the attitudes of parents of children without ASD is inconclusive. 

First, some parents have favorable attitudes towards inclusion [16,27,31,36,37,38,39]. They contend that it is positive for their children to share their daily routines with peers with special needs because it allows them to develop sensitivity, accept differences, become more prosocial citizens, learn about diversity, and enhance self-emotion [16,39,40,41].

In contrast, other parents find negative consequences when including children with ASD or other kinds of disability with their neurotypical children in mainstream schools. These parents are concerned about possible negative effects on their children’s education [8,31,33,36,42], such as imitating misbehaviors [42,43], lack of individual attention from the teacher in the inclusive classroom [31], worse grades [44], or lack of good classroom management [36,43].

Apart from assessing parents’ attitudes towards inclusion, there is also growing interest in assessing their knowledge about ASD. 

First, parents of children with disabilities have been reported to show good levels of knowledge about their children’s diagnoses [45,46]. This is a key issue because they are the primary caregivers and can notice some initial symptoms [47,48]. In this regard, knowledge means empowerment, and it is necessary to improve feelings of self-confidence about ASD in families [47,49].

However, in the case of the general population, misconceptions about ASD have been reported [11,50,51,52]. For this reason, it can be assumed that parents of children with typical development also have misconceptions. Whereas some previous studies have reported high levels of knowledge [3,41,53], other studies have confirmed insufficient levels of knowledge in this population [54].

### 1.3. Purpose of the Study

Previous literature has studied the attitudes and knowledge of families of children with ASD about this disorder. However, to our knowledge, these two variables are not usually studied together in families. In addition, the attitudes of the parents of children without ASD have not received much attention, even though they are a key factor in inclusive education because their children can play an important role in the inclusion of children with ASD. After all, inclusive education is beneficial for all the students in the classroom.

The current study aims to fill a gap in the scientific literature by investigating the attitudes towards inclusion and knowledge about ASD in parents of children with and without ASD. In addition, we take into account whether or not these children with typical development have had previous contact with a peer with ASD.

This study’s objectives were threefold.

1. To assess attitudes towards the inclusion of children with ASD in mainstream classrooms in three groups of parents: parents of children with ASD (ASD-FAM group, hereinafter), parents of children without ASD with previous or current contact with peers with ASD (PEER-CONTACT group, hereinafter), and parents of children without ASD with no prior or current contact with ASD partners (NO-PEER-CONTACT group, hereinafter).
2. To assess knowledge about ASD in the three groups of parents: ASD-FAM, PEER-CONTACT, and NO-PEER-CONTACT.
3. To compare attitudes and knowledge about ASD in these three groups of parents: ASD-FAM, PEER-CONTACT, and NO-PEER-CONTACT.

## 2. Materials and Methods

### 2.1. Research Design

**Figure 1:**
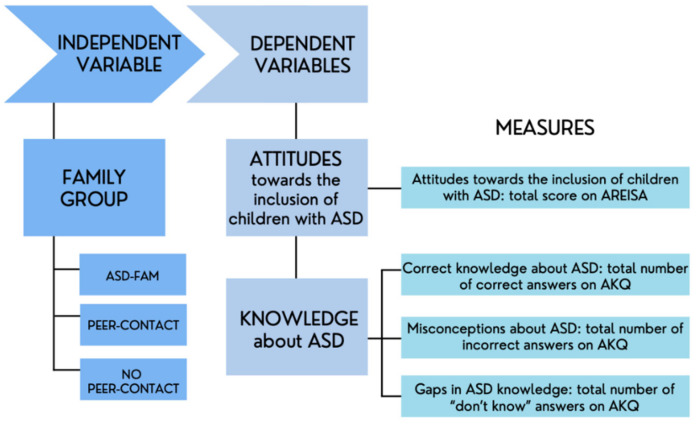
Flow chart describing the transversal unifactorial between-subject design.

The present study adopted a transversal unifactorial design that included an independent between-subject variable (family group) with three levels: ASD-FAM group, PEER-CONTACT group, and NO-PEER-CONTACT group. The dependent variables used were the measures derived from the scale of attitudes towards the inclusion of children with ASD in mainstream schools and from the ASD knowledge scale. Figure 1 shows the independent and the dependent variables and the measures used in each case.

### 2.2. Participants

A total of 170 parents, divided into three groups, participated in the study: the ASD-FAM group, composed of 75 parents of children with ASD; the PEER-CONTACT group, composed of 44 parents of children without ASD with previous or current contact with peers with ASD; and the NO-PEER-CONTACT group, composed of 51 parents of children without ASD with no prior or current contact with ASD partners. Table 1 describes the main characteristics of the sample.

### 2.3. Measures

#### 2.3.1. Attitudes Questionnaire

The Attitudes of Regular Educators Towards Inclusion of Students with Autism Survey (AREISA) [55] was translated into Spanish. The content was adapted to measure families’ attitudes towards the inclusion of children with ASD. This scale contains 22 items with which participants are expected to indicate their agreement or disagreement on a 4-point Likert scale: 1 “strongly disagree”, 2 “disagree”, 3 “agree”, and 4 “strongly agree”. A pilot test was conducted with five parents of children with ASD and five parents of children without ASD. These parents were asked to identify possible doubts or confusing aspects, and the appropriate adaptations were made based on the feedback they provided.

To check the scale’s reliability, the Cronbach alpha index was calculated, obtaining a value of 0.94, which indicates that it is an internally consistent instrument to evaluate ASD attitudes. To determine its validity, exploratory factor analysis was performed. The KMO test obtained a value of 0.93, and the Bartlett sphericity test obtained a chi-square value of 2395.89 (*p* < 0.001). The factorial structure of the scale was found to contain four factors that explained 67.7% of the total variance: Factor 1, related to implications of inclusion for students with ASD (as an illustration, *“A student with autism included in the regular classroom will display academic gains as a result of being included”*)*;* Factor 2, related to implications of inclusion for students without ASD (for instance, *“Including children with ASD in an ordinary classroom positively impacts the academic performance of other peers”*); Factor 3, related to administrative and organizational aspects of inclusion (such as “*The school principal and the educational administration of the center where it is enrolled my child should promote the philosophy that students with special educational needs are the responsibility of all* *the staff working in the school”*); and Factor 4, related to families’ self-perceived skills to promote inclusion (for example, “*I believe I can work effectively with other families to meet and satisfy the needs of children with ASD*”). We used the total score obtained on the scale in the current study.

#### 2.3.2. Autism Knowledge Questionnaire

The AKQ [56] contains 30 items that assess general knowledge about the characteristics of children with autism and how children with this disorder function. The items include three possible answers, given that they are true/false statements with a “don’t know” option, making it possible to assess not only knowledge (through the correct answers) and misconceptions (through the incorrect answers), but also possible gaps (through the “don’t know” option). This last response option (“don’t know”) keeps participants from trying to guess the correct answer, which occurs when only two choices (true/false) are offered. As an example of a true statement, we introduce “*Autism is a neurodevelopmental disorder*”, while an example of a false statement is “*With proper intervention, most children with autism spectrum disorder will eventually outgrow the disorder*”.

We used an adapted and translated Spanish version [57]. In the present study, three items whose response could be considered problematic were removed. These three items did not fully adapt to the true/false response format because they were only partially correct statements. We calculated the Cronbach alpha index for this 27-item scale version, obtaining a value of 0.91, which indicates that it is an internally consistent instrument to evaluate ASD knowledge. We used the measures of correct knowledge (total number of correct answers), misconceptions or wrong knowledge (total number of incorrect answers), and gaps (total number of “don’t know” answers). 

#### 2.3.3. Demographic Information Questionnaire

The authors developed a questionnaire to ask families about their socio-demographic information, whether or not they had children with ASD, and whether their children without ASD had previous contact with peers with ASD. Non-identifying questions about the person filling out the survey were included.

### 2.4. Data Collection Procedure

To contact families, we requested the support of 30 teachers from schools with and without communication and language classrooms. Currently, there are about 80 of these specific classrooms in the Valencian Community (a region in Spain with a population of 5 million). In these classrooms, between 7 and 10 children with ASD spend part of the school day with other children with ASD, and they spend the rest of the school day in their regular classrooms with students without ASD. Thus, we emailed these 30 teachers, attaching a link to the questionnaires on Google Forms. Teachers sent it to the families of their students in the schools with and without communication and language classrooms via email and social networks, thus involving families whose children have or do not have contact with peers with ASD, as well as families of children with ASD enrolled in these specific classrooms. These 30 teachers were able to contact 202 people. We removed the responses of 32 participants because their children were above 18 years. Eventually, we analyzed the responses offered by 170 people. 

### 2.5. Data Analysis Procedure

Analyses were performed with the SPSS statistical package, version 26 for Windows. First, to check if there were significant associations between the variables that characterize the sample, according to the family group variable, Pearson chi-square tests were carried out for the variables gender, educational level, environment where they live, marital status, family structure, economic level, and type of occupation, according to the family group variable. Kruskal–Wallis tests for independent samples and pairwise comparisons were also carried out for the variables age and number of children in the household, depending on the family group variable, adjusting the significance values with the Bonferroni correction for multiple testing. Nonparametric statistics were used after verifying that the data did not meet the assumption of normality. Second, the means and standard deviations were calculated for each of the measures of attitudes towards inclusion, correct knowledge about ASD, misconceptions about ASD, and gaps in ASD. Then, to analyze whether there were differences among the three groups of families on each of these four measures, Kruskal—Wallis tests for independent samples and pairwise comparisons were conducted, adjusting the significance values with the Bonferroni correction for multiple testing. The eta square value was also calculated to determine the size of the effect.

To make understanding the study easier, we defined some acronyms about labels used throughout the manuscript. Table 2 shows them.

## 3. Results

First, the results obtained for the Pearson chi-square statistic for each of the variables that characterize the sample, according to the family group variable, were not statistically significant in any case. All Cramer’s V values scored below 0.3. Regarding the age and number of children in the household variables, the Kruskal–Wallis H statistic was only significant for the number of children in the household variable (H = 11.202; *p* = 0.004). The pairwise comparisons showed a higher number of children in the home in PEER-CONTACT group families than in the other two-family groups (ASD-FAM group and NO-PEER-CONTACT group), where there were no differences.

Second, descriptive statistics calculated for the attitudes toward inclusion, correct knowledge about ASD, misconceptions about ASD, and gaps in ASD measures are shown in Table 3.

The results obtained for the Kruskal–Wallis H statistic to compare the attitudes towards inclusion, correct knowledge about ASD, misconceptions about ASD, and gaps in ASD measures among the three family groups were statistically significant in all cases, except for misconceptions about ASD (see Table 4). The pairwise comparisons showed that the ASD-FAM group scored significantly higher than the other two groups on pro-inclusion attitudes. In addition, the ASD-FAM group showed more accurate knowledge about ASD and fewer gaps in ASD knowledge than the other two groups. We did not find any statistically significant differences between families of children without ASD.

## 4. Discussion

The purpose of our study was threefold. We assessed families’ attitudes towards the inclusion of children with ASD in mainstream classrooms and their knowledge about the disorder, and we compared the results obtained by families of children with ASD (ASD-FAM) and families of children without ASD, taking into account whether the children without ASD had (or had not) attended classes with children with ASD (PEER-CONTACT and NO-PEER CONTACT families, respectively).

### 4.1. Families’ Attitudes towards ASD

We found that the ASD-FAM group has positive attitudes towards the inclusion of children with ASD in the mainstream schooling system. Their attitudes are more favorable than those of parents of children with typical development, coinciding with some previous studies [8,33,34]. These results are not surprising because ASD-FAM are interested in creating an inclusive society where their children are not held back and can develop their skills [29,33,34,35]. However, even in this group, few parents hold that their children should enroll in a special education school, as prior literature has contended [16]. They argue that their children would be treated more appropriately in these specific environments, with more resources, as prior literature has confirmed [29,30,31,34]. In any case, the number of parents who support special education for their children is not significant, and it is related to the severity of the characteristics of their particular child.

Families of children without ASD also showed supportive attitudes towards the inclusion of children with the disorder, although these attitudes were less favorable than those of ASD-FAM. This result coincides with previous studies [16,27,31,36,37,38,39].

First, it is logical that parents of children without ASD show lower positive attitudes towards inclusion than the families of children with the disorder. They may feel that the educational system is not ready to manage classrooms with children with and without ASD at the same time, with different specific needs. Therefore, they might be worried about the impact on their children’s academic outcomes [29,31,43].

Second, although there were differences in attitudes of ASD-FAM, according to the results obtained, the other two groups of surveyed parents also had a positive view of inclusion. This supportive perspective could be due to the fact that these parents see the benefits of inclusive education. None of the participants from the PEER-CONTACT group stated that the experience has been negative. They may think that sharing a class with a peer with ASD could be valuable to their children [16,39,40,41]. Only three parents from the NO-PEER-CONTACT group are supportive with the idea of segregated schooling for children with ASD. This may be due to the stigma derived from a lack of knowledge about ASD [54].

### 4.2. Families’ Knowledge about ASD

Regarding parents’ knowledge, in general, the scores obtained showed that all the participants had a high level of knowledge, as previous literature has found in general population surveys [4,5,11,53,58]. ASD-FAM reported higher knowledge scores than families of children without ASD. As in the attitudes scores, no significant differences were found between PEER-CONTACT and NO-PEER-CONTACT families.

ASD-FAM showed fewer gaps than the other two groups of participants. This could be related to the fact that parents of children with ASD are worried about their child’s diagnosis. They tend to be involved in finding out more and more about autism, and they try to help with their children’s development, reaching out to experts from the time the child is young. The fact that they learn about ASD and how to manage it reduces family stress and improves family routines, parental well-being, and the behavior of children with autism [59,60]. As good practice, it is now recommended that families participate in ASD education and management training programs [59]. Consequently, the ASD-FAM involved in this study shows high levels of knowledge about the disorder, few mistakes, and few gaps, which is quite positive, because parents can detect initial symptoms [25].

In the case of families of children without ASD, they also have a good level of knowledge about ASD, although less than the parents of children with ASD, which agrees with results obtained in previous studies [3,41,53]. These conclusions may be related to the increase in inclusive policies in our country and, specifically in our region, the Valencian Community. This situation may affect parents’ attitudes about enrolling their children in inclusive schools, as well as their levels of knowledge about ASD. The high level of knowledge in families of children without ASD does not coincide with a recent study conducted in Pakistan [54]. This disparity in the results could be explained by cultural factors related to the presence of ASD in the media in the two environments. In recent years, civil organizations in Spain have made intense efforts to disseminate knowledge and awareness campaigns about ASD in the population [61], and they have had an impact in the mass media. This may have contributed to an increase in the Spanish population’s knowledge about ASD.

### 4.3. Inclusion of Children with ASD

The general scores in attitudes and knowledge obtained in this study present a very positive outlook for the future of inclusive education because parents have positive attitudes towards the inclusion of children with ASD, and this could probably influence their children’s attitudes in a welcoming and inclusive way [13,14,22,25]. It is known that knowledge and parental attitudes are not the only factor influencing their children’s stigma. Children’s peers of the same age is also a variable that should be taken into account, to name but one, and not only the attitudes of parents. However, the fact that parents involved in this study show favorable attitudes towards inclusive education could be the first step to help to eliminate stigma in peers of children with ASD [9,20,21].

There are no differences between the attitudes and knowledge of families of children without ASD depending on whether their children have had previous contact with a peer with the disorder. The fact that parents of children without ASD also display positive attitudes towards inclusion could be rooted in an emerging and growing awareness disseminated by some communication media, ASD associations, and inclusive policies. The media and social networks broadcast audiovisual products and content that, in many cases, favor the visibility of disability. Thus, disorders such as ASD are more well known, and stigma and prejudice can be reduced [62]. These phenomena make it possible for society to show better attitudes towards the inclusion of people with autism and improve and increase their knowledge about the disorder.

Inclusive policies also mean that children with disabilities are included in schools, sharing experiences with non-disabled children, and providing employment opportunities for adults with disabilities who can work alongside other non-disabled adults. All these facts may help to create a non-discriminatory image of ASD.

### 4.4. Practical Implications

The results of this study may have some practical implications for schools where students with ASD are enrolled. The fact that there are no differences in attitudes and knowledge about ASD between PEER-CONTACT and NO-PEER-CONTACT families suggests that schooling a child with ASD is a phenomenon that practically goes unnoticed within the family. This is a positive point because it seems to indicate that the problematic situations of rejection or stigmatization identified in some previous studies were not found [9,63,64]. However, this outcome also seems to suggest that including a child with ASD provides an opportunity for children without ASD to be enriched by the experience of attending inclusive schools with students with other conditions, such as a diagnosis of autism.

Therefore, we believe it would be advisable for schools to enroll students with ASD (as occurs in the majority of state schools in the Spanish education system), carrying out specific activities where the richness of having students with different conditions is valued. These events could be held, for example, on World Autism Awareness Day or International Asperger’s Syndrome Day. These specific awareness-raising activities, which require a moderate effort on the part of teachers, could be good opportunities to positively impact the entire community by providing children and families with basic information about ASD. This could help the whole community, including parents, to form even more positive attitudes towards ASD, as suggested and recommended in previous literature [65,66].

### 4.5. Study Limitations and Recommendations for Future Research

We would like to point out some limitations of the study. 

First, limitations to sample size must be pointed out. In addition, there is a cultural bias, since the variables under investigation are likely highly context-specific and based on many factors, likely to vary quite significantly depending on the group’s embedded cultural factors. Thus, the lack of generalizability of the results should be taken into account when reading our results. Secondly, the instruments used only provide quantitative data. In the specific case of the attitudes survey, it is probably bound to social desirability bias, which makes it difficult to delve into an increasingly entrenched problem. 

Regarding some recommendations for future research, they are related to the mentioned limitations.

Future studies in the same line should analyze the same variables in a larger sample and compare populations in different locations to avoid sample size limitations and cultural bias. It would also be advisable to analyze qualitative aspects with appropriate instruments in order to provide better insight into the results. Regarding future lines of research, it would also be interesting to study implicit attitudes, and it might be advisable to perform some type of intervention that would contribute to families’ positive attitudes towards ASD. Another area that might be considered in future studies and seems overlooked is assessing child beliefs and understanding how these are congruent or incongruent when compared to the parents’ ones.

## 5. Conclusions

Parents are important agents because they educate their children, who may or may not have autism spectrum disorder. The current study showed that parents of children with and without ASD have positive attitudes towards this disorder and correct knowledge about it. We did not find significant differences between PEER-CONTACT and NO-PEER-CONTACT families of children without ASD. This is not alarming because, in this case, both groups have good attitudes and appropriate knowledge. However, this result may suggest that we are missing the opportunity to highlight the educational value of students with different characteristics who are attending the same class. Families are a key factor in inclusive education because their children can play an important role in the inclusion of other children with ASD. It is necessary to continue to learn and break down prejudices to achieve real inclusion. Sharing inclusive classrooms could be the first step. Children who enroll in inclusive schools are the future citizens of our society. This reality may help to promote a more respectful society where diversity is treated as a natural condition.

## Figures and Tables

**Table 1:** Demographic Data of the Parents.

Characteristics	Options	ASD-FAM ^1^ (*n* = 75)	PEER-CONTACT ^1^ Families (*n* = 44)	NO-PEER-CONTACT ^1^ Families (*n* = 51)
Gender [*n* (%)]	Male	9 (12%)	4 (9.09%)	7 (13.73%)
	Female	66 (88%)	40 (90.91%)	44 (86.27%)
Age (*M* ^1^; *SD* ^1^)		42.34 (5.87)	42.36 (5.86)	43.90 (5.94)
Number of children (*M* ^1^; *SD* ^1^)		1.79 (0.81)	2.25 (0.89)	1.82 (0.97)
Educational level [*n* (%)]	Primary/basic	3 (4.05%)	1 (2.27%)	4 (7.84%)
	Mid-level	19 (25.33%)	9 (20.45%)	7 (13.73%)
	High-level	53 (70.67%)	34 (77.27%)	40 (78.43%)
Environment where they live [*n* (%)]	Rural	10 (13.33%)	4 (9.09%)	7 (13.73%)
	Urban	65 (86.67%)	40 (90.91%)	44 (86.27%)
Marital Status [*n* (%)]	Married/in a couple	68 (90.67%)	37 (84.09%)	45 (88.24%)
	Single	3 (4%)	2 (4.55%)	2 (3.92%)
	Separated	4 (5.33%)	5 (11.36%)	4 (7.84%)
Family Structure [*n* (%)]	Nuclear family	63 (84%)	35 (79.55%)	42 (82.35%)
	Remarried family	5 (6.67%)	2 (4.55%)	3 (5.88%)
	Single-parent family	6 (8%)	6 (13.64%)	5 (9.80%)
	Extended family	1 (1.33%)	1 (2.27%)	1 (1.96%)
Economic level [*n* (%)]	<1000	5 (6.67%)	2 (4.55%)	0 (0%)
	1000–2000	33 (44%)	17 (38.64%)	12 (23.53%)
	>2000	37 (49.33%)	25 (56.82%)	39 (76.47%)
Occupation [*n* (%)]	Full-time job	36 (48%)	30 (6.82%)	36 (70.59%)
	Part-time job	15 (20%)	7 (15.91%)	9 (17.65%)
	Homemaker	16 (21.33%)	5 (11.36%)	2 (3.92%)
	Unemployed	7 (9.33%)	0 (0%)	3 (5.88%)
	Retiree or Pensioner	1 (1.33%)	0 (0%)	0 (0%)
	Student	0 (0%)	2 (4.55%)	1 (1.96%)

^1^ *M*: mean; *SD*: standard deviation; ASD-FAM group: parents of children with ASD; PEER-CONTACT group: children without ASD with previous or current contact with peers with ASD; NO-PEER-CONTACT group: parents of children without ASD with no prior or current contact with ASD partners.

**Table 2:** Acronyms used throughout the manuscript.

Acronyms	Description
ASD	Autism Spectrum Disorder
ASD-FAM	It refers to the group of parents of children with ASD
PEER-CONTACT/PEER	It refers to the group of parents of typically developing children with prior or current access to children with ASD
NO-PEER-CONTACT/ NO-PEER	It refers to the group of parents of typically developing children without access to children with ASD

**Table 3:** Descriptive statistics for groups.

Descriptive Statics	Groups	*n*	*M* ^1^	*SD* ^1^
Total score attitudes	ASD-FAM	75	78.60	8.76
	PEER-CONTACT	44	70.82	11.18
	NO-PEER-CONTACT	51	70.84	13.33
Total number of correct answers on AKQ	ASD-FAM	75	21.51	3.57
	PEER-CONTACT	44	15.75	5.37
	NO-PEER-CONTACT	51	15.37	6.25
Total number of misconceptions on AKQ	ASD-FAM	75	2.99	2.28
	PEER-CONTACT	44	3.45	2.27
	NO-PEER-CONTACT	51	3.61	2.73
Total number of gaps on AKQ	ASD-FAM	75	2.51	2.84
	PEER-CONTACT	44	7.80	5.97
	NO-PEER-CONTACT	51	8.02	6.97

^1^ *M*: mean; *SD*: standard deviation.

**Table 4:** Average Range (AR) and Kruskal–Wallis H-test statistic values obtained for measures of attitudes towards inclusion, correct knowledge about ASD, misconceptions about ASD, and gaps in ASD knowledge, depending on the family group variable.

Variables	AR ASD-FAM (*n* = 75)	AR PEER-CONTACT (*n* = 44)	AR NO-PEER-CONTACT (*n* = 51)	Kruskal-Wallis *Hp*	*p*	*η2H*	Group Differences
Attitudes towards ASD	103.93	68.24	73.28	19.107 **	<0.001	0.102	ASD-FAM > PEER, NO-PEER
Correct Knowledge	114.38	62.57	62.81	46.378 **	<0.001	0.265	ASD-FAM > PEER, NO-PEER
Misconceptions about ASD	79.13	91.14	90.01	2.310	0.315	0.001	-
Gaps in ASD knowledge	59.69	108.11	103.94	37.572 **	<0.001	0.213	ASD-FAM < PEER, NO-PEER

** *p* < 0.01; “-” means “no significant differences”.

## Data Availability

The data presented in this study are available on request from the corresponding author.
